# Supplementary material for: Identification of photoperiod-induced specific miRNAs in the adrenal glands of Sunite sheep (Ovis aries)
Source: Front Vet Sci. 2022 Jul 22;9:888207. doi: 10.3389/fvets.2022.888207 (PMC9354845; doi:10.3389/fvets.2022.888207)
Supplement: Supplementary file 1 [file Data_Sheet_1.ZIP › Supplementary file/miRDeep2 file.zip!/miRDeep2 file/Know_Structure/SPLP42A2/oar-mir-376b.pdf]

miRBase precursor : oar-mir-376b
Total read count : 482
oar-miR-376e-5p read count : 193
oar-miR-376b-5p read count : 193
oar-miR-376b-3p read count : 96
remaining reads : 0

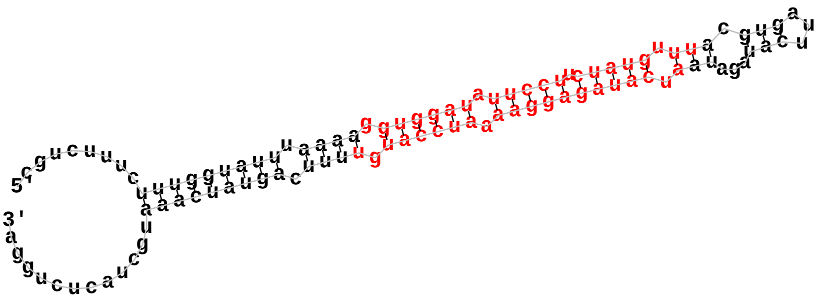

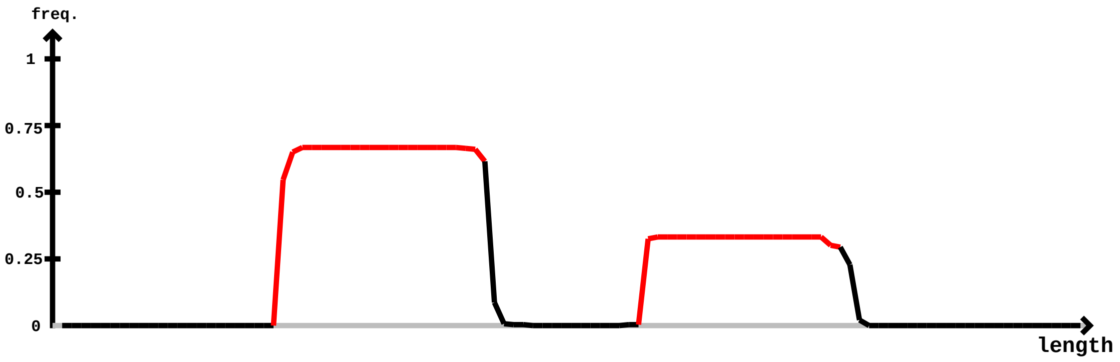


```
                                                          oar-miR-376b-3p
                      oar-miR-376b-5p
                      oar-miR-376e-5p
5'- cgucuuucuuugguauuuaaaagguggauauuccuucuauguuuacgugauucauagauaaucauagaggaaaauccauguuuucaguaucaaaugcuacucugga  -3'   exp
    ........(((((((((.((((.((((((.(((((.(((((.(((.(((...)))...))).)))))))))).)))))).)))).)))))))))............   reads   mm   sample
    ......................gguggauauuccuucuaug.................................................................   1       0    seq
    ......................gguggauauuccuucuaugu................................................................   1       0    seq
    ......................gguggauauuccuucuauguu...............................................................   13      0    seq
    ......................gguggauauuccuucuauguuu..............................................................   125     0    seq
    ......................gguggauauuccuucuauguuua.............................................................   18      0    seq
    .......................guggauauuccuucuauguuu..............................................................   26      0    seq
    .......................guggauauuccuucuauguuua.............................................................   4       0    seq
    ........................uggauauuccuucuauguuu..............................................................   2       0    seq
    ........................uggauauuccuucuauguuua.............................................................   1       0    seq
    ........................uggauauuccuucuauguuuac............................................................   1       0    seq
    ........................uggauauuccuucuauguuuacgu..........................................................   1       0    seq
    ..........................................................uaaucauagaggaaaauccaug..........................   1       0    seq
    ............................................................aucauagaggaaaauccau...........................   9       0    seq
    ............................................................aucauagaggaaaauccaug..........................   1       0    seq
    ............................................................aucauagaggaaaauccaugu.........................   19      0    seq
    ............................................................aucauagaggaaaauccauguu........................   60      0    seq
    ............................................................aucauagaggaaaauccauguuu.......................   4       0    seq
    .............................................................ucauagaggaaaauccauguuu.......................   2       0    seq
```
